# Twitter integration of chemistry software tools

**DOI:** 10.1186/s13321-021-00527-x

**Published:** 2021-07-02

**Authors:** Naruki Yoshikawa, Ryuichi Kubo, Kazuki Z. Yamamoto

**Affiliations:** 1 Department of Computer Science, University of Toronto, Toronto, Canada; 2 Isotope Science Center, The University of Tokyo, Tokyo, Japan; 3 SHaLX Inc., Tokyo, Japan; 4 Department of Computer Science, Tokyo Institute of Technology, Yokohama, Japan

**Keywords:** Twitter, Social media, Retrosynthesis, Visualization, Molecule editor, Chemical space, Knowledge sharing, Open science

## Abstract

Social media activity on a research article is considered to be an altmetric, a new measure to estimate research impact. Demonstrating software on Twitter is a powerful way to attract attention from a larger audience. Twitter integration of software can also lower the barriers to trying the tools and make it easier to save and share the output. We present three case studies of Twitter bots for cheminformatics: retrosynthetic analysis, 3D molecule viewer, and 2D chemical structure editor. These bots make software research more accessible to a broader range of people and facilitate the sharing of chemical knowledge, concepts, and ideas.

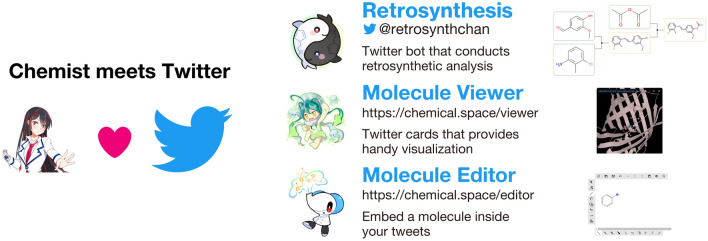

## Introduction

Social media such as Twitter has become an important communication tool for scientists. *Twitter* is a social networking service on which users post short messages called *tweet*, interact with each other by sending a *reply* and share other users’ tweets by *retweet*. Scientists are using *hashtags* such as #chemtwitter, #RealTimeChem, #ScienceTwitter, #compchem and #OpenScience to create communities on Twitter for networking and information exchange [1]. Twitter activities about a research paper are considered to be one of the altmetrics [2], which is an alternative to citation counting for measuring scientific impact. Some studies reported that the number of tweets on a research article is positively correlated to the article citations [3–5]. Attracting attention on Twitter is getting more important to promote scientific works.

A Twitter bot is an automated program on Twitter controlled through Twitter API. Bots are common on Twitter, and they usually deliver tweets like news and blog updates [6]. Twitter bots are also used for scientific purposes, such as posting updates of journal publications or preprints. While most bots automatically post tweets, they can also respond to replies from other users. We can construct an interactive application using Twitter API as long as following the rules and policies of Twitter such as Automation rules [7].

In this paper, we report case studies of cheminformatics tools demonstrated on Twitter by introducing the functionality of our Twitter bots and Twitter cards. The Twitter account @souyakuchan [8] was originally created for promoting the authors’ project of crowd-based drug discovery (named Social Drug Discovery). It has been hosting a simple cheminformatics bot since 2017 in the form of a bot that draws a 2D structural formula image for replies containing a SMILES or an IUPAC name [9]. The bot was later expanded by integrating functions in chemical.space [10], which are described in the case study 2 and 3. The second account @retrosynthchan [11] offers retrosynthesis functionality. It was implemented in December 2020 for the blog event called Drug Discovery (dry) Advent Calendar 2020 [12] hosted by @souyakuchan. We describe the retrosynthesis bot in the case study 1.

## Case study 1: retrosynthesis bot

In this section, we introduce a Twitter bot @retrosynthchan [11], which conducts retrosynthetic analysis by AiZynthFinder [13]. The bot responds to a request given as a SMILES [14] string and returns the results of retrosynthetic analysis for the given molecule. Users are expected to send a reply containing only a SMILES string right after the bot’s screen name (e.g., @retrosynthchan c1ccccc1Br). The communication between users and the bot is in public, so every Twitter user can see the query and result.

### Implementation

The workflow of the bot program is as follows: (1) retrieve a reply to the bot via Twitter API, (2) check whether the given string is valid as SMILES using RDKit [15] and send a request confirmation, (3) run retrosynthesis analysis by AiZynthFinder [13], (4) send the retrosynthesis result images via Twitter API. The details of each step will be discussed.

#### Retrieve reply

A reply in Twitter is a message toward a specific account, and it usually starts with the screen name of the receiver (e.g., @jack How are you today?). At the time of this writing, the Twitter API provides a method to retrieve tweets that contain a specific keyword in real-time (POST statuses/filter [16]). The bot receives a reply to the bot by retrieving tweets that contain the bot’s screen name with this API. A Python library Tweepy [17] is used to handle Twitter API.

#### Confirm request

After receiving a reply, the string is separated into parts by a space. The second part is assumed to be a SMILES string since the first part is the bot’s screen name. The SMILES string is considered to be valid if it can be successfully parsed by RDKit [15] with the default setting. The bot returns the input molecule’s chemical structure if the input is valid, otherwise, send back an error message to the sender and finish. The confirmation is sent as a reply to the users by a Twitter API POST statuses/update [18] using Tweepy.

#### Run retrosynthetic analysis

Retrosynthetic analysis of the molecule represented by the given SMILES is conducted by AiZynthFinder [13], open-source software for retrosynthetic planning. The input molecule is broken down into purchasable precursors with the algorithm based on a Monte Carlo tree search guided by a neural network. The synthesis route images are generated for individual routes.

#### Send synthetic pathway images

The generated synthetic route images are sent to the user as a reply with images. Twitter allows users to attach only up to four images in one post, so three images are connected into one image using Pillow [19] to send a maximum of twelve synthesis routes. The generated images are sent back to the users with the Twitter API using Tweepy.

### Results

**Fig. 1:**
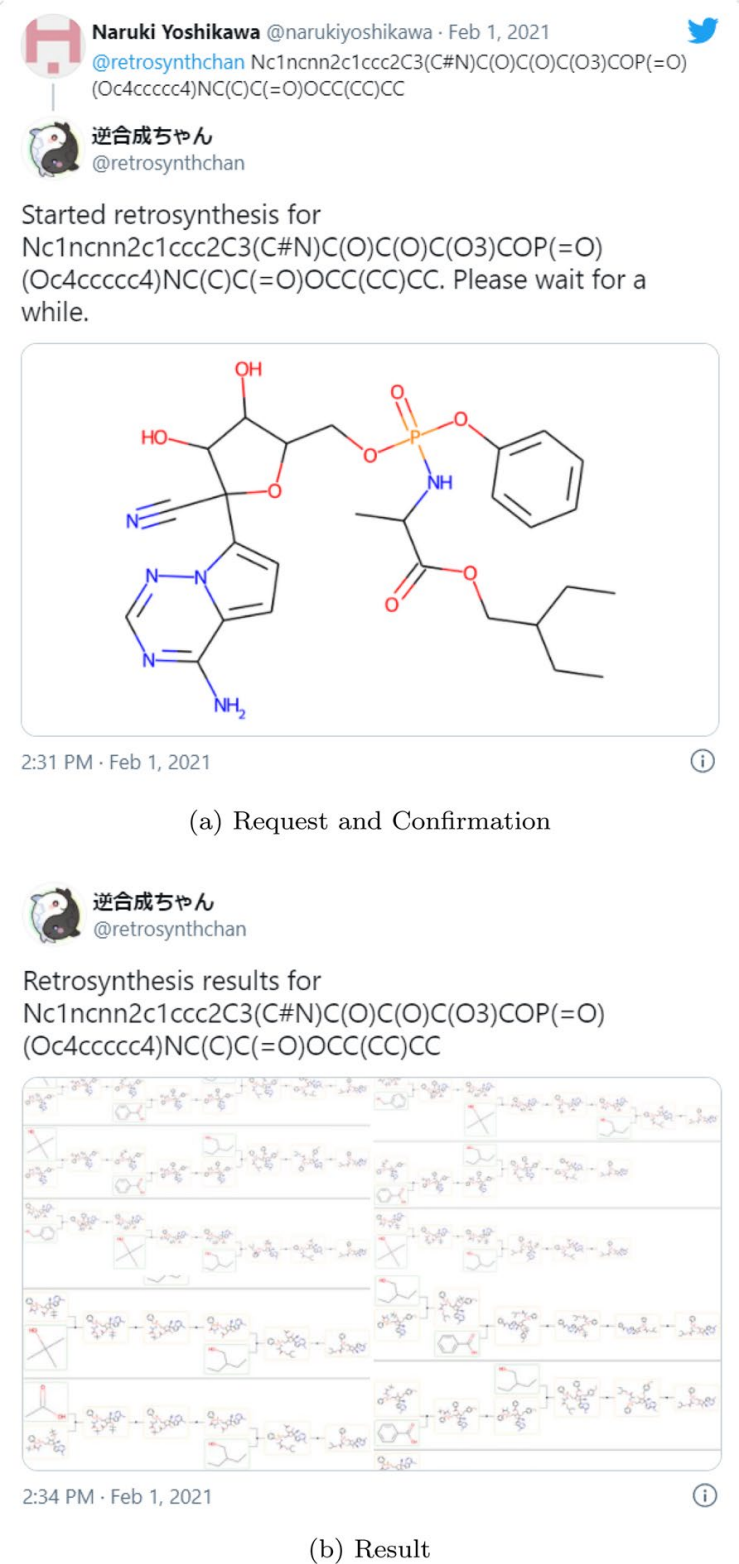
Screenshots of the retrosynthesis bot. **a** A tweet requesting the bot to run retrosynthetic analysis, and the bot’s reply confirming the request. The molecular structure image is attached in the response. **b** The bot’s reply showing the result of retrosynthesis. Four images are attached to the tweet, and each image shows three results of retrosynthesis

A screenshot of the bot in action is shown in Fig. 1. A request confirmation with a chemical structure image was sent right after the SMILES was sent to the bot. The result of the retrosynthetic analysis is sent several minutes later since it takes a while to run AiZynthFinder. Typically, it takes less than 3 min to return the result since we use the default time limit (120 s) of AiZynthFinder, but it may be delayed if other people are running the job.

The bot attracted attention from Twitter users interested in chemistry, and it gained more than one hundred followers in the first 2 weeks. The followers include chemists in academia, researchers in the drug industry, undergraduate or graduate chemistry students, etc. The Twitter bot helped the retrosynthesis software outreach to a broader range of people.

## Case study 2: 3D molecule viewer embedded in Twitter timeline

Next, we introduce a 3D molecule viewer which can be played on a Twitter timeline inlinely via our web server chemical.space [10]. This feature is based on the Twitter Player Card system [20]. The Player Card system is used as a video/audio player in the Twitter timeline, wherein the 3D molecule viewer works as interactive video content. This feature is currently available on web browsers, while the Twitter mobile application can not load Player Card directly in the timeline due to the limitation of Twitter’s specifications. Users can view any Protein Data Bank (PDB) structures interactively without leaving their timeline on the browser. To embed the viewer in a tweet, users are expected to post a tweet containing a PDB ID string right after the bot’s screen name with a #pdb hashtag (e.g., @souyakuchan 1ema #pdb).

### Implementation

As a 3D molecule viewer, we adopted LiteMol [21], which is a JavaScript application written in TypeScript. The way of receiving requests is the same as in Case 1. After receiving the PDB ID via users’ tweets, the bot makes a reply in which the URL of our hosting server of LiteMol is indicated. LiteMol refers to the URL parameter and fetches the PDB data. Also, to provide Twitter Player Card, our viewer web page serves meta information in HTML header named twitter:card. Twitter Player Card can render HTML referring an URL in the tweet. The meta information is mainly utilized for recognizing the player (embedded web page) URL and the player title.

### Results

**Fig. 2:**
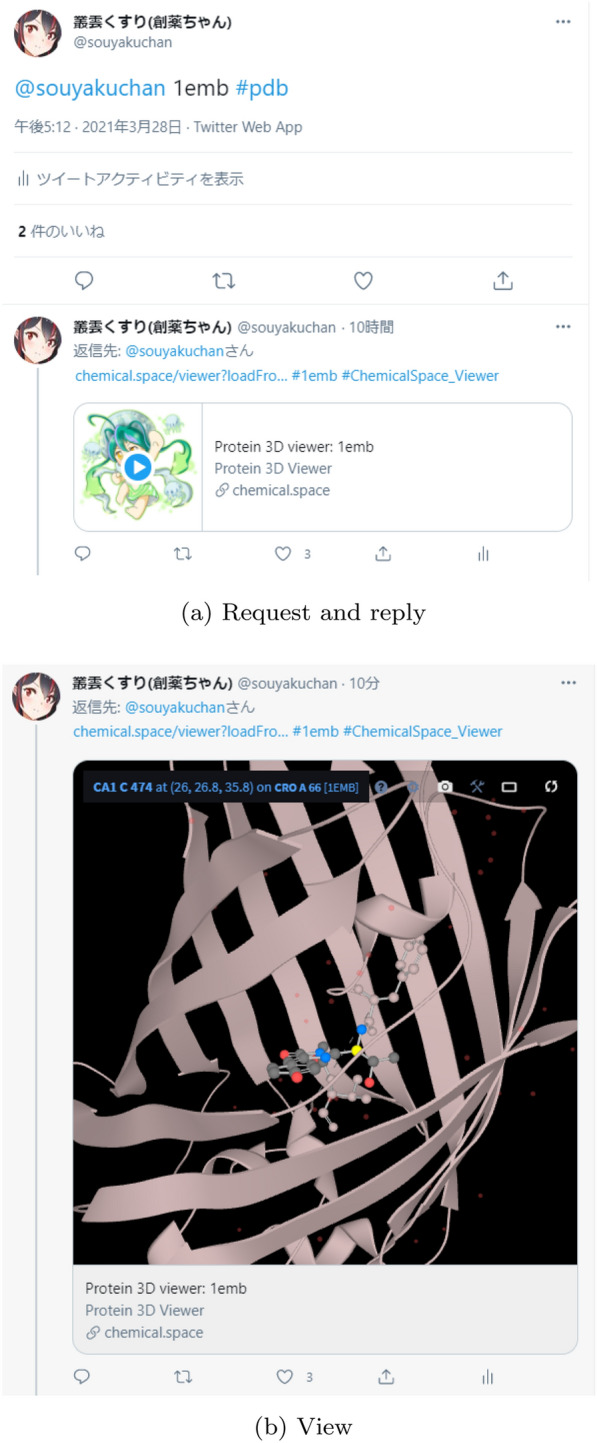
Screenshots of the Twitter integrated 3D molecule viewer. **a** A tweet requesting the bot to return an URL of a 3D molecular viewer and the subsequent reply with the viewer embedded in the Twitter timeline. Users can expand the viewer by clicking the play button. **b** The viewer expanded in the Twitter timeline. Users can interactively manipulate the view

A screenshot of the viewer played on a Twitter timeline is shown in Fig. 2. After receiving a PDB code by a *mention* tweet, the bot immediately replies with an URL in which the 3D viewer Player Card can be expanded. Users can view the 3D structure interactively as it is in the Twitter timeline.

## Case study 3: shareable 2D chemical structural formula editor

Finally, we introduce a Twitter Player Card which offers an easy way to share a molecule inside a tweet. The 2D chemical structural formula editor integrated with tweet posting function (available at https://chemical.space/editor) is one of the easiest ways to share one’s own idea of chemical structures on Twitter. Users can share their manually edited structural formula as a SMILES or Chemical Markup Language (CML) [22] format. By using CML, users can include the information of coordinate and rotation of each object. This is a better way to share complex structures, whereas SMILES can only represent the molecular graph information, which has the advantage of the smallest data size. This editor has one more useful feature, in which users can send their manually drawn chemical structure to @retrosynthchan bot (shown in Case Study 1) by just clicking the RETROSYNTHESIZE button.

### Implementation

Among the existing well-designed traditional browser-based 2D molecule editors such as JSME [23] and MarvinSketch [24], we chose Kekule.js [25] for its ease of integration as a web application and its sufficient functionality. Its JavaScript-based editor can input and output SMILES strings and CML strings of manually drawn structural formulas. Also, we used rdkit.js [26] to convert SMILES query to Molfile format [27] containing 3D coordinates. While SMILES only stores simple characters to express atoms, bonds, rings, and their orders, CML has coordinate and rotation information of each atom and bond. Furthermore, CML contains many XML markup tags in addition to that information. Meanwhile, CML strings become too long to tweet because one tweet accepts only 280 characters. However, URL strings are not included in this limitation. To embed CML strings into an URL, https://chemical.space/editor compresses CML strings by gzip using pako [28] library, then encodes it into Base64url using brianloveswords/base64url [29].

### Results

**Fig. 3:**
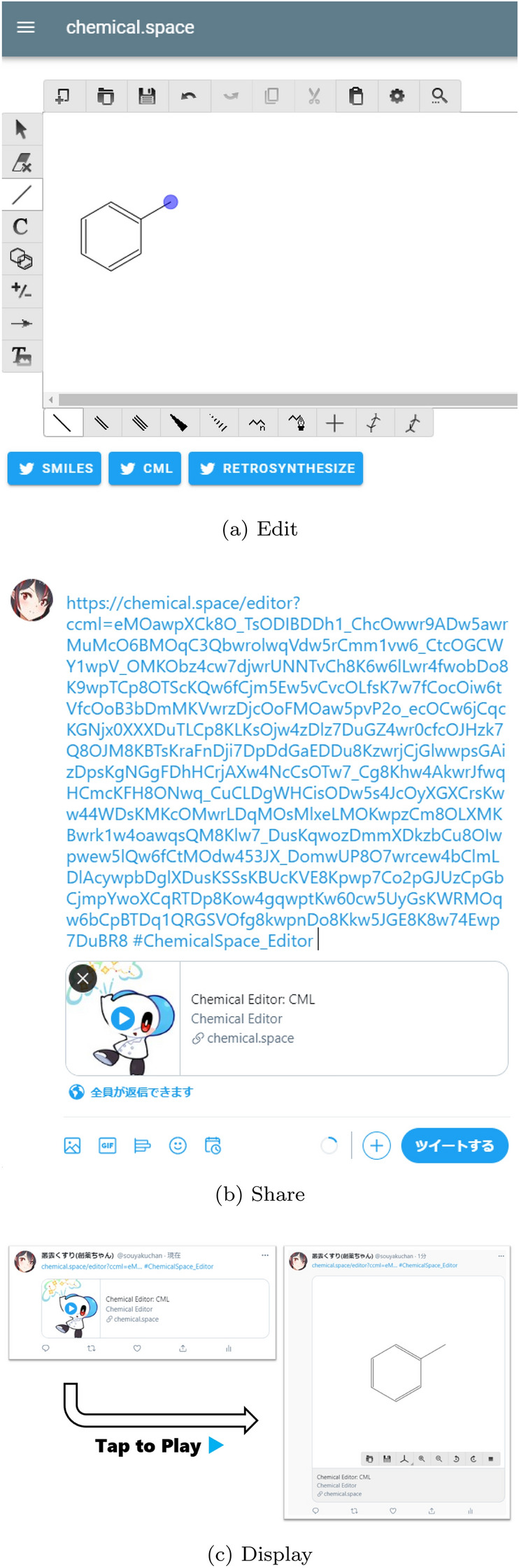
Screenshots of the Twitter-integrated 2D editor. **a** The 2D molecule editor integrated with tweet buttons. **b** The tweet input window to share the manually drawn structural formula. The CML strings are encoded in the URL. **c** The 2D molecule viewer expanded on the Twitter timeline. At the moment, intra-timeline player is only supported on the PC browser version of Twitter, and not on the mobile App version

The 2D molecule editor hosted on https://chemical.space/editor is shown in Fig. 3. Users can utilize it to draw structural formulas from scratch in their browser and post them to Twitter in SMILES or CML format using the button at the bottom of the editor. We have also prepared a button that allows users to submit their structural formula to the retrosynthetic analysis bot introduced in Case Study 1. Structural formulas posted in CML format can be re-edited by opening the URL in the tweet, which is useful for open discussion on compounds on Twitter.

## Discussion

As presented in these case studies, it is technically possible to make any cheminformatics tool accessible and noticeable from social networking services in the form of interfacing with Twitter bots. However, to make effective impressions on the scientific community and stimulate discussion, it is necessary to use appropriate hashtags that are widely prevailing and be tweeted by accounts with a large number of followers and strong influence. It would be helpful for scientists to follow accounts of mutual interest regularly and grow their follow/follower count appropriately. Tweets with high-impact and sensational content are likely to generate more impressions, but the information described in the tweet must have a certain level of accuracy. As with the sanitization of structural formulas [30], it is also beneficial for peers of scientists to ensure each other’s scientific soundness on the Twitter timeline. An attempt to socially share ideas for compounds has also emerged very recently in the fight against the pandemic coronaviral disease COVID-19 (e.g., the COVID Moonshot project [31–33]), which is an example of the trend toward open science in drug discovery. Looking ahead to applications in drug discovery, we also need widgets in which users can interactively view and discuss the interaction and activity of compounds with target proteins, which is a future challenge. As a semi-permanently maintained database infrastructure, Twitter has the potential to store an unlimited amount of chemical information.

## Conclusions

We presented three examples of Twitter integrated cheminformatics tools in this study. Twitter integration can offer a convenient interface to run cheminformatics software without a laborious environment setup, and users can easily share the result by tweet and retweet. Providing a demonstration on social media will be a new approach to present one’s research and stimulate discussion in a more accessible manner. If you would like to add your own tool to our bot functions, please contact the authors for support.

## Availability and requirements

Case study 1

- Project name: Twitter Retrosynthesis Bot
- Project home page: https://github.com/n-yoshikawa/retrosynthesis_bot
- Operating system(s): Platform independent
- Programming languages: Python
- Other requirements: AiZynthFinder, RDKit, Tweepy, Pillow
- License: MIT License

Case study 2 and 3

- Project name: chemical.space
- Project home page: https://github.com/ddquest/chemical.space
- Operating system(s): Platform independent
- Programming languages: Python
- Other requirements: LiteMol, RDKit, Tweepy
- License: MIT License

## Data Availability

The source codes of Case 1 software are available at﻿ 10.5281/zenodo.4999041. The source codes of the Case 2 and 3 software are available at﻿ 10.5281/zenodo.4979985. The chemical.space applications are available at https://chemical.space/.
